# 不同极性固定相上气相色谱保留指数机器学习集成预测模型的构建

**DOI:** 10.3724/SP.J.1123.2024.07014

**Published:** 2025-04-08

**Authors:** Qianyi WANG, Yongle ZHU, Xuehua LI

**Affiliations:** 大连理工大学环境学院,工业生态与环境工程教育部重点实验室,辽宁 大连 116024; Key Laboratory of Industrial Ecology and Environmental Engineering, Ministry of Education, School of Environmental Science and Technology, Dalian University of Technology, Dalian 116024, China

**Keywords:** 气相色谱保留指数, 集成学习, 不同极性固定相, McReynolds常数, gas-chromatographic retention index, ensemble learning, different polar stationary phases, McReynolds’ constant

## Abstract

保留指数是在色谱分析中用于表征化合物保留性能的指标,是用于化合物结构鉴定的重要参数。化合物在不同极性固定相上的保留指数差异,使得当前基于单一极性固定相的保留指数预测模型无法有效应用于多种极性固定相的保留指数预测。因此,本研究建立了不同极性固定相上气相色谱保留指数预测模型,从文献中收集到2499种化合物在8种类型固定相上的保留指数数据共4183条,根据McReynolds常数进一步将固定相划分为强极性、极性、中等极性、弱极性与非极性五类,耦合化合物分子结构特征与固定相极性独热编码特征作为模型输入,采用9种算法构建了机器学习预测模型。基于模型性能最优的XGBoost和LightGBM算法,采用投票回归建立集成学习模型,其训练集决定系数(*R*^2^)为0.99,训练集均方根误差(RMSE)为101.85,测试集*R*^2^为0.97,测试集RMSE为107.44。采用Williams图表征模型的应用域,有94%以上的数据在应用域内。本研究综合固定相极性和化合物结构两类复合特征,成功开发了能够适应多种极性固定相的保留指数预测模型,克服了现有单一极性固定相模型的局限性,极大地拓宽了模型的应用范围。与个体机器学习模型相比,集成模型体现出了更好的稳健性和预测能力。模型的建立对于提高气相色谱靶标和非靶标分析的效率和准确性具有重要的科学意义和实际价值。

## Abstract (en)

Gas chromatography is an analytical technique that is widely used to separate and identify various compounds. The retention index (RI) plays a significant role in gas chromatography because it provides a standardized measure for characterizing the retention performance of compounds under specific conditions and is a powerful compound-identification tool, particularly when dealing with complex mixtures. Consequently, the ability to predict RI values is a meaningful objective, particularly for multipolar phases, owing to significant variations in RI across various polar stationary phases. To address this issue, we developed a model for predicting gas-chromatographic RIs on stationary phases of varying polarity by collecting 4183 pieces of retention-index data for 2499 compounds on eight types of stationary phase from the literature and databases. Stationary phases were further classified into five categories based on their the McReynolds constants, namely: strongly polar, polar, medium polar, weakly polar, and non-polar. This classification ensured that the model is capable of handling a wide range of polarities, thereby enhancing its versatility and applicability to various analytical scenarios. The predictive model was constructed by integrating two types of composite feature. The 1D and 2D molecular-structural features of the compounds were first determined; these features capture the chemical and physical properties of the compounds, including their relative molecular masses, functional groups, and topological indices. These descriptors provide a comprehensive understanding of the molecular characteristics that influence retention behavior. Stationary-phase polarity was then one-hot encoded, which converted categorical stationary-phase-polarity information into a format that can be effectively used by machine-learning algorithms. This encoding technique ensures that the model can distinguish among the effects of various polarities on the retention behavior of the compounds. Nine algorithms were used to construct predictive machine-learning models, including linear regression, decision tree, random forest, support vector machine (SVM), k-nearest-neighbor (KNN), gradient-boosting decision tree (GBDT), extreme gradient boosting (XGBoost), and light gradient boosting (LightGBM) algorithms. Voting regression was used to build an optimally performing ensemble learning model based on the XGBoost and LightGBM algorithms. This ensemble model, which combines the strengths of multiple individual models, exhibited exceptional performance, with a training set coefficient of determination (*R*^2^) of 0.99, a training set root mean square error (RMSE) of 101.85, a test set *R*^2^ of 0.97, and a test set RMSE of 107.44. Williams plots were used to characterize the application domain of the model, with over 94% of the data lying within the domain, indicative of broad applicability and high predictive confidence. The successful development of this predictive retention-index model represents a significant advancement in the gas-chromatography field. The developed model offers several key benefits by integrating advanced machine learning techniques with comprehensive chemical- and physical-property data; it highly accurately predicts RI values across a wide range of polar stationary phases. The developed ensemble model exhibits superior robustness and predictive abilities compared to individual machine-learning models. The establishment of this model is of great scientific significance and practical value for improving the efficiency and accuracy of target and non-target gas-chromatographic analyses.

气相色谱法是利用气体作为流动相的一种色谱分析方法,化合物从进样至色谱柱后出现浓度极大值时所需的时间定义为保留时间。不同实验室或不同色谱条件下,同一种化合物的保留时间存在差异,一般无法通用^[1]^。将不同色谱分析方法所得的保留时间归一化为与系统无关的常数,称为保留指数(retention index, RI), RI不仅可用于化合物定性分析,还可用于关联、计算或估算其他理化参数,如沸点、蒸汽压、碳数、物质的量等^[2]^。RI理论上只和化合物本身的性质和色谱柱固定相有关,不受实验室条件的影响,这就使得不同实验室间RI数据具有可比性,进而可以利用RI对化合物进行识别和鉴定。

目前,已有研究致力于建立化合物分子结构与RI之间的关系模型。近5年来,研究者主要依据一类或特定领域的物质如醛酮化合物、烷烃、饱和酯、辣椒成分、精油成分等在指定色谱柱下的RI数据建立模型。Anjum等^[3]^和Qu等^[4]^基于美国国家标准与技术研究院(NIST)数据库采用图神经网络方法构建预测模型,数据量达到十万余条,模型决定系数(*R*^2^)在0.90以上,但是建模原始数据未公开。其他相关研究^[5⇓⇓⇓⇓⇓⇓-12]^的RI数据基本均从文献或实验中获得,建模数据量在数百条左右,且现有模型很少考虑到不同极性固定相对RI预测的影响。此外,现有模型对杀虫剂、多环芳烃等环境类化合物RI值预测十分有限。鉴于其具有的持久性、生物蓄积性和毒性等特点,建立环境类化合物的高效检测和预测模型具有极其重要的现实意义。

因此,本研究全面收集了文献和数据库中已报道的RI数据,包括环境污染物、香气化合物等多领域的化合物,基于McReynolds常数将色谱柱分为强极性、极性、中等极性、弱极性和非极性5种类别并进行独热编码处理,融合5种色谱柱极性特征和化合物分子结构信息,作为模型的特征输入,采用9种机器学习算法构建RI集成预测模型。

## 1 实验与方法

### 1.1 数据集

本研究根据McReynolds常数值来判断固定相的极性,它通过实验测得。1970年,McReynolds^[13]^采用苯、丁醇、2-戊酮、硝基丙烷、吡啶这5种标准物质,在恒定120 ℃的柱温条件下,系统地计算了226种不同的固定相与角鲨烷之间的RI差值Δ*I*,取这5种物质的平均Δ*I*值,即可得到McReynolds常数,具体由公式(1)计算得到,*I*表示物质的RI。

本研究根据McReynolds常数值来判断固定相的极性,它通过实验测得。1970年,McReynolds^[13]^采用苯、丁醇、2-戊酮、硝基丙烷、吡啶这5种标准物质,在恒定120 ℃的柱温条件下,系统地计算了226种不同的固定相与角鲨烷之间的RI差值Δ*I*,取这5种物质的平均Δ*I*值,即可得到McReynolds常数,具体由公式(1)计算得到,*I*表示物质的RI。


(1) $$\text{McReynolds 常数}=\frac{1}{5}\sum_{i=1}^{5}\left(I_{i}^{\text{固定相}}-I_{i}^{\text{参照物}}\right)$$

根据McReynolds提出的极性分级体系,McReynolds常数低于100的固定相定义为非极性固定相,100~400的固定相定义为中等极性固定相,而大于400的固定相则定义为强极性固定相^[13]^。McReynolds常数数值越大,说明固定相的极性越强。实验过程中可以通过McReynolds常数选择与样品性质相匹配的固定相,以优化气相色谱的分离效果。

本研究对此进行更细致的划分,McReynolds常数为0~50的固定相为非极性固定相,50~100的固定相为弱极性固定相,100~250的固定相为中等极性固定相,250~400的固定相为极性固定相,大于400的固定相为强极性固定相。气相色谱主要用于挥发和半挥发性有机化合物的分析,本研究从文献中收集了烃类(链烃和环烃)及其衍生物(醛、酮、酯、醇、酚、醚、胺、酰胺、腈类等)的4183条RI数据,共2499种化合物,且覆盖8种类型色谱柱(carbowax-20M、DB-225MS、DB-624、OV17、DB-5、HP5-MS、HP-1、OV101),每个极性区间内均有对应极性色谱柱的RI数据,构成最终的RI数据集,如表1所示。

**表1:** 4237条建模数据来源及分布情况

Category	Number	Chromatographic column	Stationary phase	McReynolds’ constant	Refs.
Strong polarity	1372	carbowax-20M	polyethylene glycol	462	[14-18]
Polarity	198	DB-225MS	50% cyanopropylphenyl-50% dimethylpolysiloxane	363^*^	[19]
Medium polarity	484	DB-624	6% cyanopropylphenyl-94% dimethylpolysiloxane	158^*^	[20]
		OV17	50% diphenyl-50% dimethylpolysiloxane	177	[14]
Weak polarity	1316	DB-5	5% diphenyl-95% dimethylpolysiloxane	67	[14,21]
		HP5-MS		67	[19]
		-		67	[15,16]
Non-polar	817	HP-1	100% dimethylpolysiloxane	44	[19]
		OV101		44	[14]
		-		44	[15,16,22]

-: The column type is not given in the literature, but the composition of the stationary phase is known. * McReynolds’constant was obtained from a similar stationary phase.

### 1.2 数据预处理

获取数据集中化合物的简化分子线性输入规范(simplified molecular input line entry system, SMILES),将其转化为smi格式,将smi文件输入至PaDEL-Descriptor软件中,获得化合物1维和2维分子结构特征共1444个。设置方差阈值为0,以过滤冗余特征,删除皮尔逊相关系数大于0.9的多重共线性特征。为消除特征之间的差异性,对连续值特征进行标准化处理。采用递归特征消除法进行特征选择,剔除不重要特征,最终得到20个化合物的结构特征。

对5种不同极性的色谱柱进行独热编码,得到5个定性特征强极性、极性、中等极性、弱极性和非极性,将其与定量特征耦合,作为模型的特征输入。

### 1.3 机器学习模型构建与优化

将上述20个特征作为最终的特征输入,RI值作为模型的预测终点,构建不同极性固定相上化合物分子结构与RI的机器学习预测模型。按7∶3的比例将数据集随机拆分为训练集和测试集,进行多次划分,绘制不同随机种子(random seed)下集成模型的*R*^2^与均方误差(RMSE)的值如图1所示。本文选择random_state取值为80,此时训练集和测试集*R*^2^均最高,分别为0.99和0.97。

**图1:**
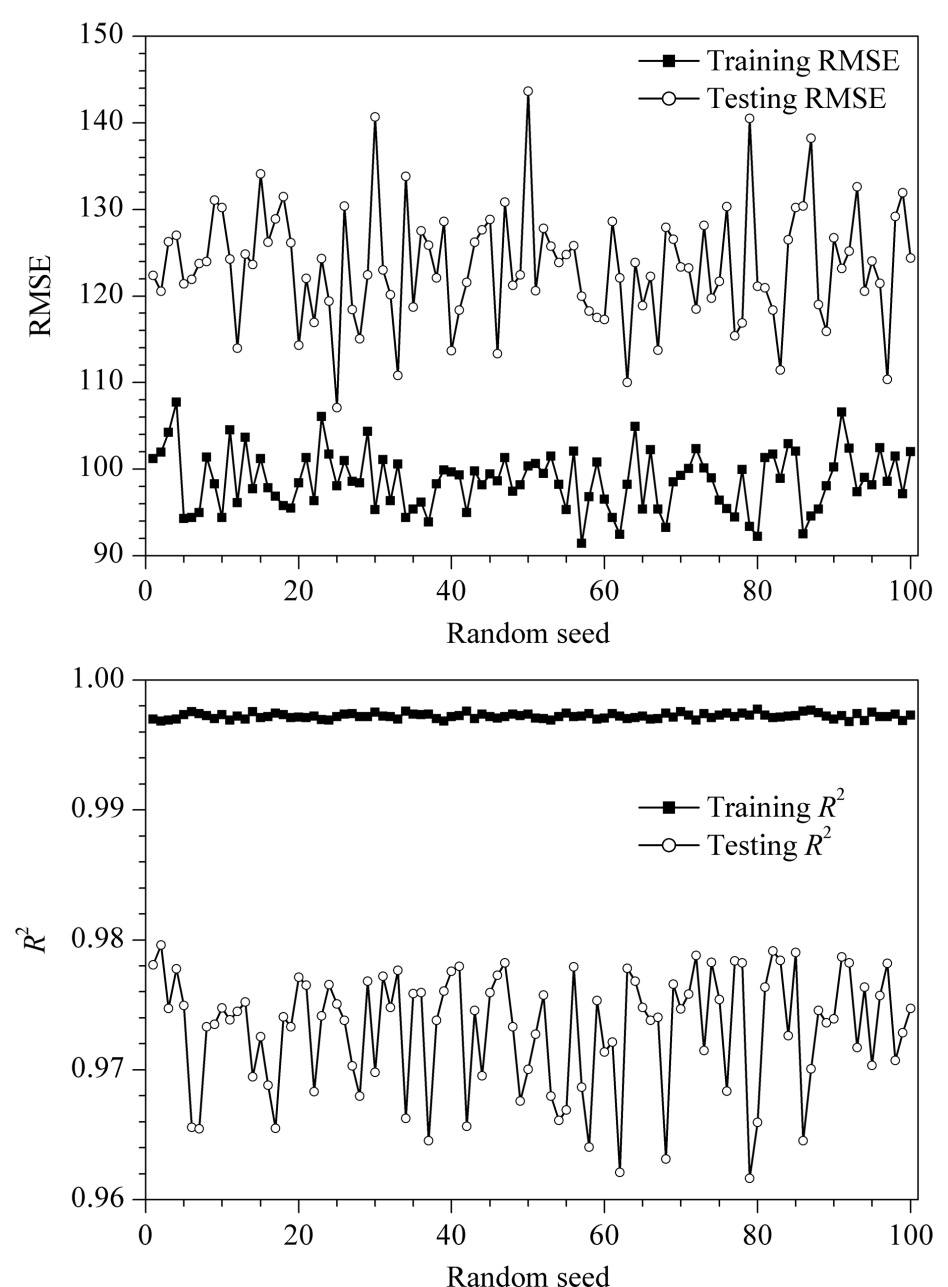
不同随机种子下(a)RMSE和(b)*R*^2^的值

采用9种机器学习算法:线性回归(LR)、决策树(DT)、随机森林(RF)、支持向量机(SVR)、K近邻(KNN)、梯度提升决策树(GBDT)、极致梯度提升(XGBoost)、自适应增强(AdaBoost)、轻量梯度提升(LightGBM)构建模型,在训练集上进行网格搜索循环遍历寻找模型的最佳超参数,如图2所示。使用10折交叉验证来评估不同超参数的效果,即训练集被进一步划分为10个子集,每个子集轮流作为验证集,剩余9个子集作为训练集来训练模型。使用*R*^2^作为判断指标,选择使*R*^2^值最优的超参数作为最终的超参数。

**图2:**
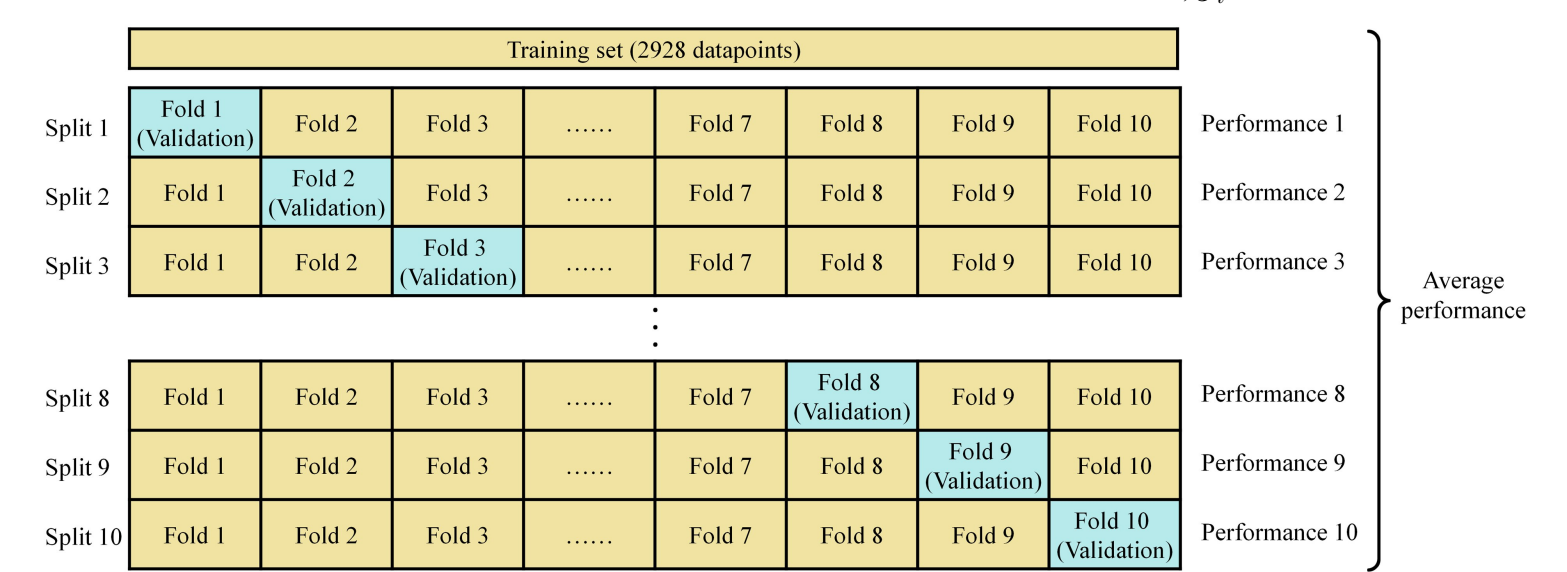
10折交叉验证进行超参数选择的流程图

将模型性能较优的算法作为基学习器,建立投票回归(VR)预测模型,预测结果是所有模型预测结果的平均值,最佳超参数源于每个基学习器的最佳超参数。

### 1.4 模型评估

*R*^2^是回归模型的拟合度量,其取值范围为[0,^1^]。一般来说,*R*^2^越大,表示模型拟合效果越好。RMSE表示预测值与真实值之间的平均偏差程度,RMSE越小表示模型的预测越准确。

训练集交叉验证系数(cross-validation coefficients, $Q_{CV}^{2}$ )用于判断模型的稳健性,由公式(2)计算,${{\hat{y}}_{i}}^{\left(D-M\right)}$ 为*D-M*个样本所得到的模型对被剔除的第*i*个样本终点的预测值,${{\overset{-}{y}}_{i}}^{\left(D-M,i\right)}$ 为*D-M*个样本实测值的平均值,*y_i_*为第*i*个样本点的真实

值,*n*为样本数。如果$Q_{CV}^{2}$ 大于0.60,模型比较稳健,如果$Q_{CV}^{2}$ 大于0.90,模型的稳健性非常好。


(2) $$Q_{CV}^{2}=1-\frac{\sum_{i=1}^{n}{\left({\hat{y}}_{i}^{\left(D-M\right)}-y_{i}\right)}^{2}}{\sum_{i=1}^{n}{\left(y_{i}-{\bar{y}}_{i}^{\left(D-M,i\right)}\right)}^{2}}$$

测试集外部验证系数(externally validated determination coefficient, $Q_{ext}^{2}$ )用于评价模型的预测能力。由公式(3)计算,${\hat{y}}_{i}$ 为第*i*个样本点的预测值,${\overset{-}{y}}_{i}$ 是实测值的平均值,*n*为样本数。一般认为,$Q_{ext}^{2}$ 大于0.50,模型预测能力比较好;$Q_{ext}^{2}$ 大于0.80,模型的预测能力非常好。


(3) $$Q_{ext}^{2}=1-\frac{\sum_{i=1}^{n}{\left(y_{i}-{\hat{y}}_{i}\right)}^{2}}{\sum_{i=1}^{n}{\left(y_{i}-{\bar{y}}_{i}\right)}^{2}}$$

### 1.5 应用域表征

基于标准化残差和杠杆距离*h_i_*绘制Williams图,表征投票回归模型的应用域,其中训练集特征包括分子结构特征和色谱柱极性特征,若化合物的杠杆距离*h_i_*大于杠杆距离警戒值h^*^,则认为该化合物在应用域之外。

*h_i_*和h^*^的计算公式如式(4)~(6)所示,*X*为训练集中化合物和特征的矩阵,*H*为帽子矩阵,[*H*]*_ii_*为矩阵*H*对角线值,*p*为训练集特征的个数。


(4) *H*=*X*(*X*^T^*X*)^-1^*X*^T^

(5) *h_i_*=[*H*]*_ii_*

(6) $$h^{*}=\frac{3(p+1)}{n}$$

## 2 结果与讨论

### 2.1 数据分析

2499种化合物中有45种化合物在5类固定相上均有RI数据,使用单因素方差分析法检验这45种化合物的RI在不同极性固定相上是否存在显著差异^[23]^。如图3所示,极性由弱到强依次为非极性、弱极性、中等极性、极性、强极性。a、b两组RI数据之间有显著性差异(*p*<0.05),固定相变化造成RI变化的现象不是偶然。

**图3:**
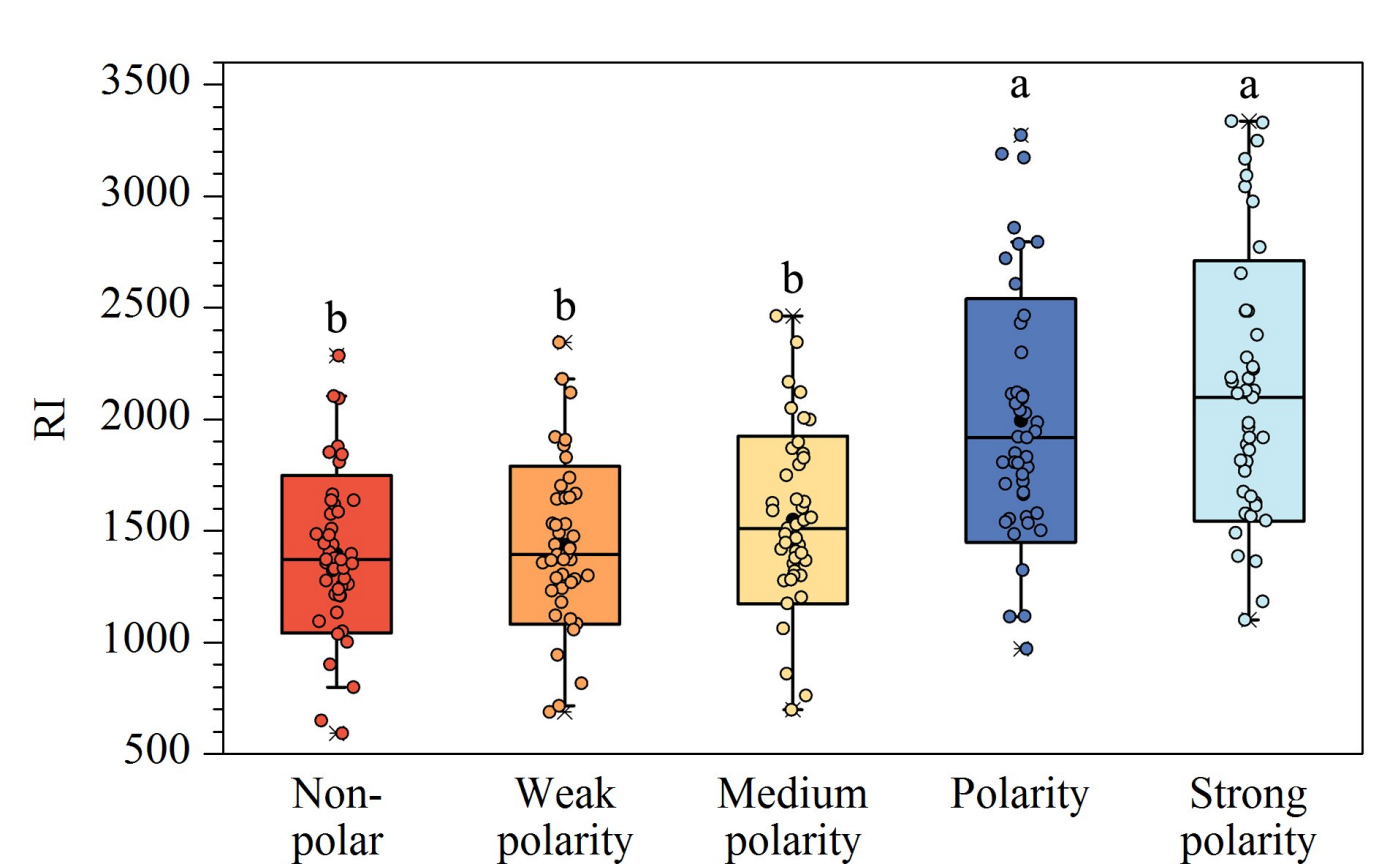
不同极性色谱柱下化合物RI单因素方差分析(*n*=45)

### 2.2 特征选择和机器学习模型构建

经过方差过滤,保留1206个特征;经过皮尔逊相关系数过滤,保留475个特征;经过递归特征消除法,最终选择20个分子结构特征与5个极性哑变量特征输入模型。分子结构特征包括自相关(ATS1m、ATS1s、ATSC1e、AATSC1v、ATSC1p)、负荷修正特征值(SpMin8_Bhe、SpMax2_Bhv、SpMax2_Bhs、SpMax3_Bhs)、分子线性自由能关系(MLFER_S、MLFER_E)、价键轨道Chi path(AVP-6、ASP-6)、原子极化率之和(apol)、Barysz矩阵(VR3_DzZ、VE3_DzZ)、扩展拓扑化学原子(ETA_Shape_P)、拓扑电荷(GGI6)。基于随机森林特征重要性排序如图4所示,其中ATS1m、ATS1s、MLFER_E、Strong polar、MLFER_S是前5个重要的特征。

**图4:**
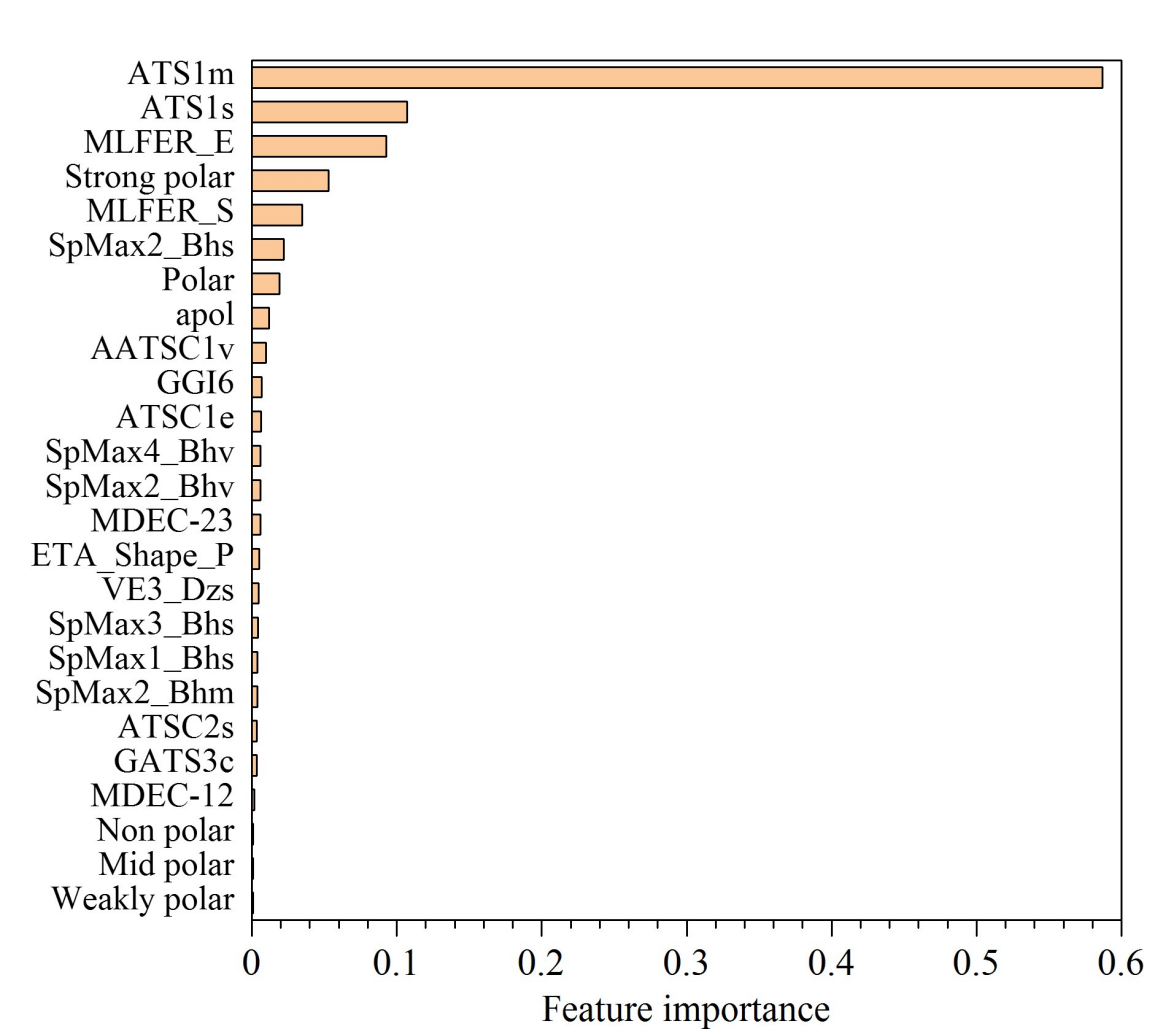
随机森林特征重要性排序

机器学习模型的超参数及模型性能如表2和表3所示,投票回归预测模型训练集和测试集RI拟合图如图5所示。综合考虑训练集和测试集的$Q_{cv}^{2}$ 、$Q_{ext}^{2}$ 、*R*^2^和RMSE可以发现,LR和SVM整体相比不如其他复杂模型;DT、RF和KNN在训练集上预测准确,但测试集误差较高,模型的泛化能力待提升;GBDT、XGBoost、AdaBoost和LightGBM为梯度提升模型,通过不断叠加众多简单模型,每步重点修正上一步的预测错误,均展示出高预测能力和稳健性。本研究最终选择以XGBoost和LightGBM为基学习器建立投票回归预测模型。训练集$Q_{cv}^{2}$ 达0.97,RMSE为101.85;测试集$Q_{ext}^{2}$ 达0.97,RMSE为107.44。投票回归模型通过集成XGBoost和LightGBM的优势,在保持预测准确度的同时,训练集和测试集的RMSE分别降低了5.00和7.69,显著提高了基学习器中一些预测误差较大化合物的预测结果,从而进一步提高预测模型的稳健性和准确度。

**表2:** 10种机器学习模型的超参数

Regression model	Hyperparameterization
LR	-
DT	max_depth=17.00, random_state=85.00, min_
	samples_leaf=1.00, min_samples_split=4.00
RF	n_estimators=251.00, random_state=0.00,
	max_depth=17.00
SVR	kernel=radial basis function, C=49.00
KNN	n_neighbors=7.00, weights=‘distance’
GBDT	n_estimators=291.00, random_state=0.00
XGBoost	Booster=‘gbtree’, n_estimators=166.00, earning_
	rate=0.12, max_depth=5.00, colsample_bytree=0.56,
	gamma=0.99, reg_alpha=0.57, reg_lambda=0.91,
	subsample=0.90
AdaBoost	base_estimator=DecisionTreeRegressor, n_estimators=
	21.00, random_state=80.00, learning_rate=0.30
LightGBM	n_estimators=299.00, learning_rate=0.10,
	max_depth=5.00, random_state=80.00
VR	-

**表3:** 10种机器学习模型的预测性能

Regression model	Training (*n*=2928)				Testing (*n*=1255)		
	*R*^2^	$Q_{CV}^{2}$	RMSE		*R*^2^	$Q_{ext}^{2}$	RMSE
LR	0.93	0.93	163.81±14.55		0.93	0.94	153.55±12.13
DT	0.99	0.96	166.19±22.53		0.956	0.95	186.87±19.72
RF	1.00	0.93	114.31±15.32		0.92	0.91	134.78±13.52
SVR	0.88	0.86	228.68±32.04		0.87	0.77	288.76±36.38
KNN	0.99	0.92	165.20±16.84		0.91	0.90	180.24±20.13
GBDT	0.99	0.96	113.12±17.95		0.96	0.97	108.04±8.87
XGBoost	0.99	0.97	106.03±14.25		0.97	0.97	107.82±14.60
AdaBoost	0.99	0.96	116.03±18.33		0.96	0.94	143.13±18.73
LightGBM	0.99	0.97	104.67±17.19		0.97	0.96	116.94±15.77
VR	0.99	0.97	101.85±17.73		0.97	0.97	107.44±15.63

$Q_{cv}^{2}$ : cross validation coefficient; $Q_{ext}^{2}$ : externally validated determination-coefficient.

**图5:**
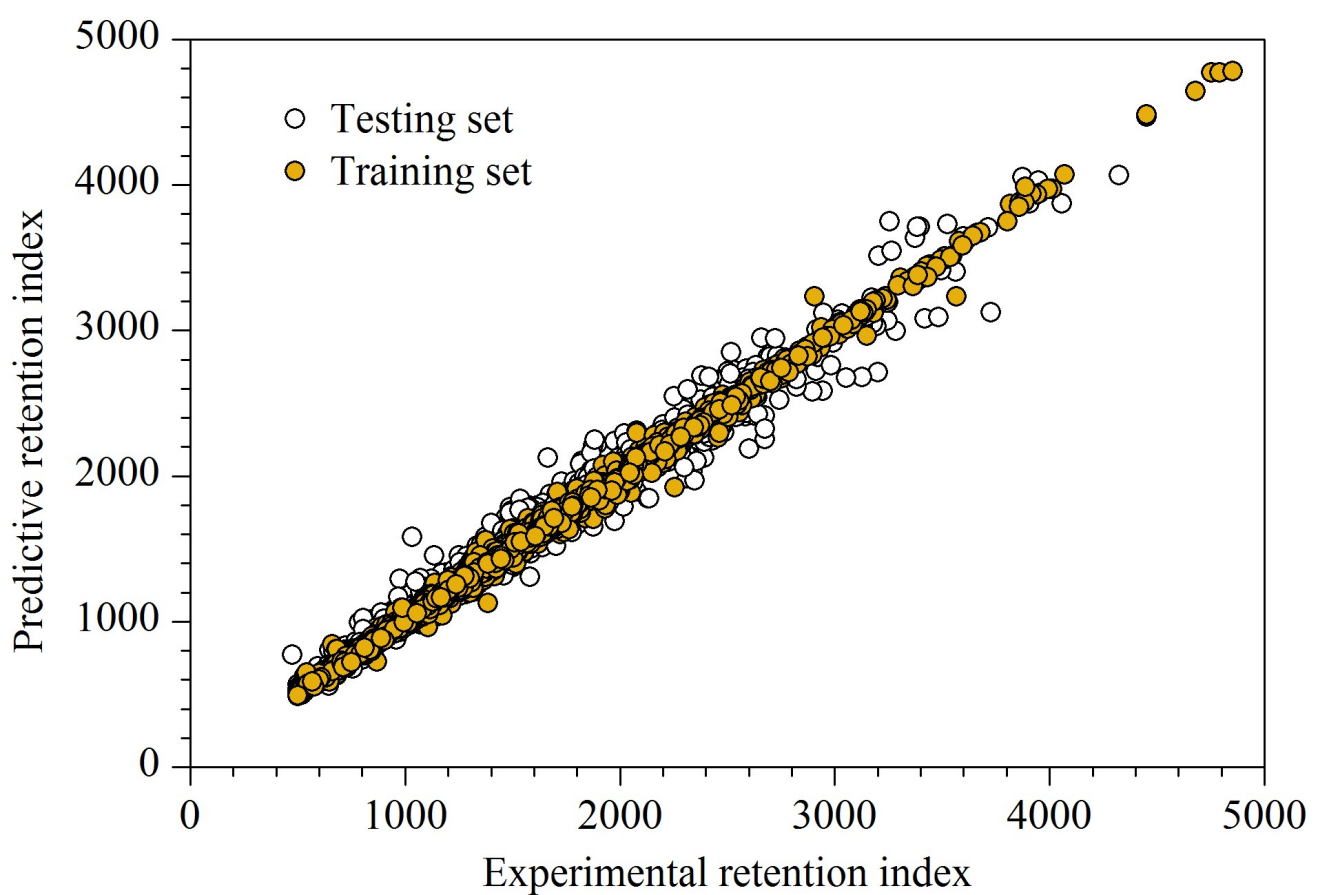
投票回归模型的预测保留指数与实验保留指数关系图

### 2.3 机理解释

分析SHAP值大于50的化合物分子结构特征如何显著影响随机森林模型的预测输出,结果如图6所示。纵轴按照所有样本的SHAP值之和对特征排序,横轴是SHAP值,即特征对模型输出的影响分布。图中每个点代表一个样本,样本量在纵向堆积,点的颜色表示特征值。特征值为低值(蓝色)时,说明该特征对RI预测是负向影响,特征值为高值(红色)时是正向影响。

**图6:**
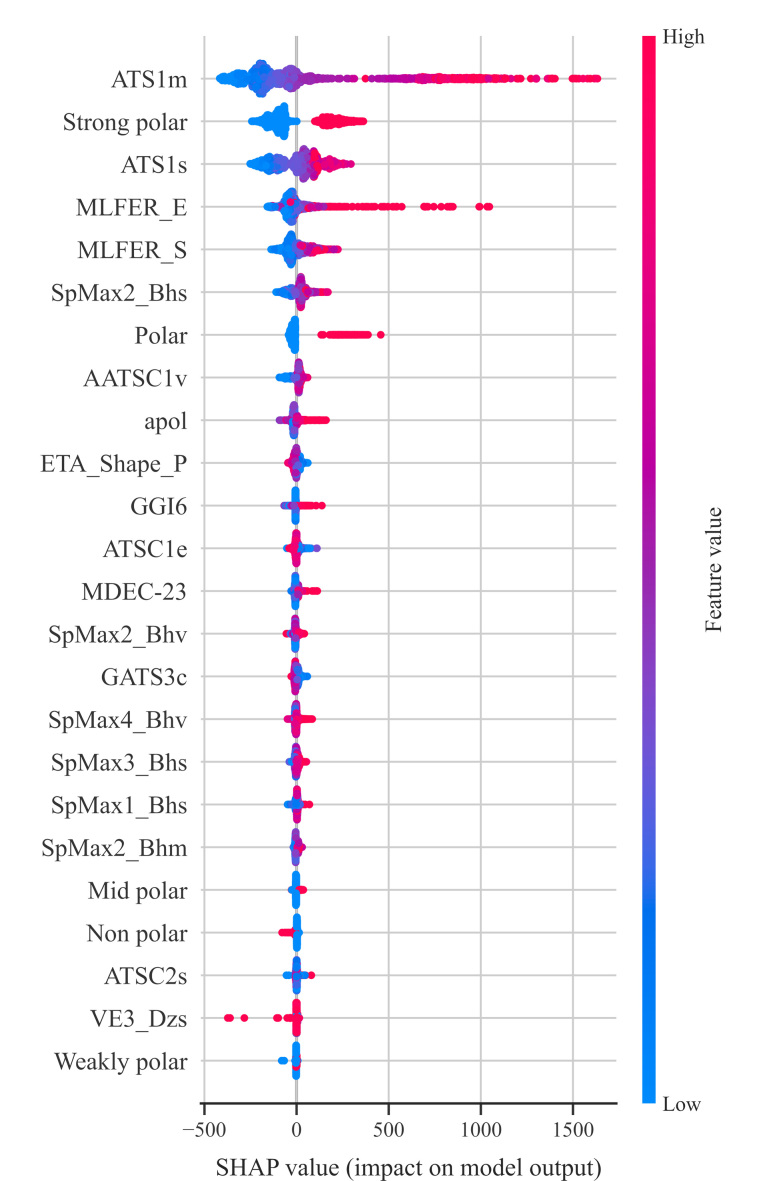
24个特征的单个SHAP值

拓扑指数是分子结构的数学描述符,用以反映分子的大小、形状、分支等结构特征,从而实现分子结构信息的数值化。拓扑结构自相关(autocorrelation of a topological structure, ATS)是拓扑指数中一种空间自相关度量方式。在Moreau-Broto自相关计算框架下^[24]^, ATS1m表示一步拓扑距离内原子的相对原子质量自相关,其值越大,意味着分子中存在较多原子(如碳、氮、氧等)紧密连接区域,使分子形成更稳定的构象。ATS1s表示一步拓扑距离内原子的疏水性自相关,其值越大,说明化合物与固定相之间有更强的疏水相互作用,从而在色谱柱中的保留时间增加,RI较高。

MLFER是分子线性自由能关系描述符^[25]^。其中,MLFER_E和MLFER_E分别与分子的极化率和摩尔折射度有关。极化率指分子电子云易被极化的程度,极化率越大,化合物与固定相相互作用力越强,RI越大。摩尔折射度反映分子偏折光线的能力,高摩尔折射度的分子通常体积更大或电子云更密集,从而减慢其在色谱柱中的行进速度,在SHAP图中表现为RI更高。

### 2.4 应用域表征

在投票回归预测模型中,杠杆距离警戒值h^*^为0.027,说明该模型适用于杠杆距离小于0.027的化合物RI的预测,杠杆距离大于警戒值的化合物均在模型的应用域之外。同时,将标准化残差的绝对值大于3的化合物认定为异常值,也归为应用域之外。如图7所示,训练集94.5%的化合物都在应用域之中,测试集95.3%的化合物都在应用域之中。且应用域之外的化合物标准化残差较小,说明模型有较好的泛化能力。

**图7:**
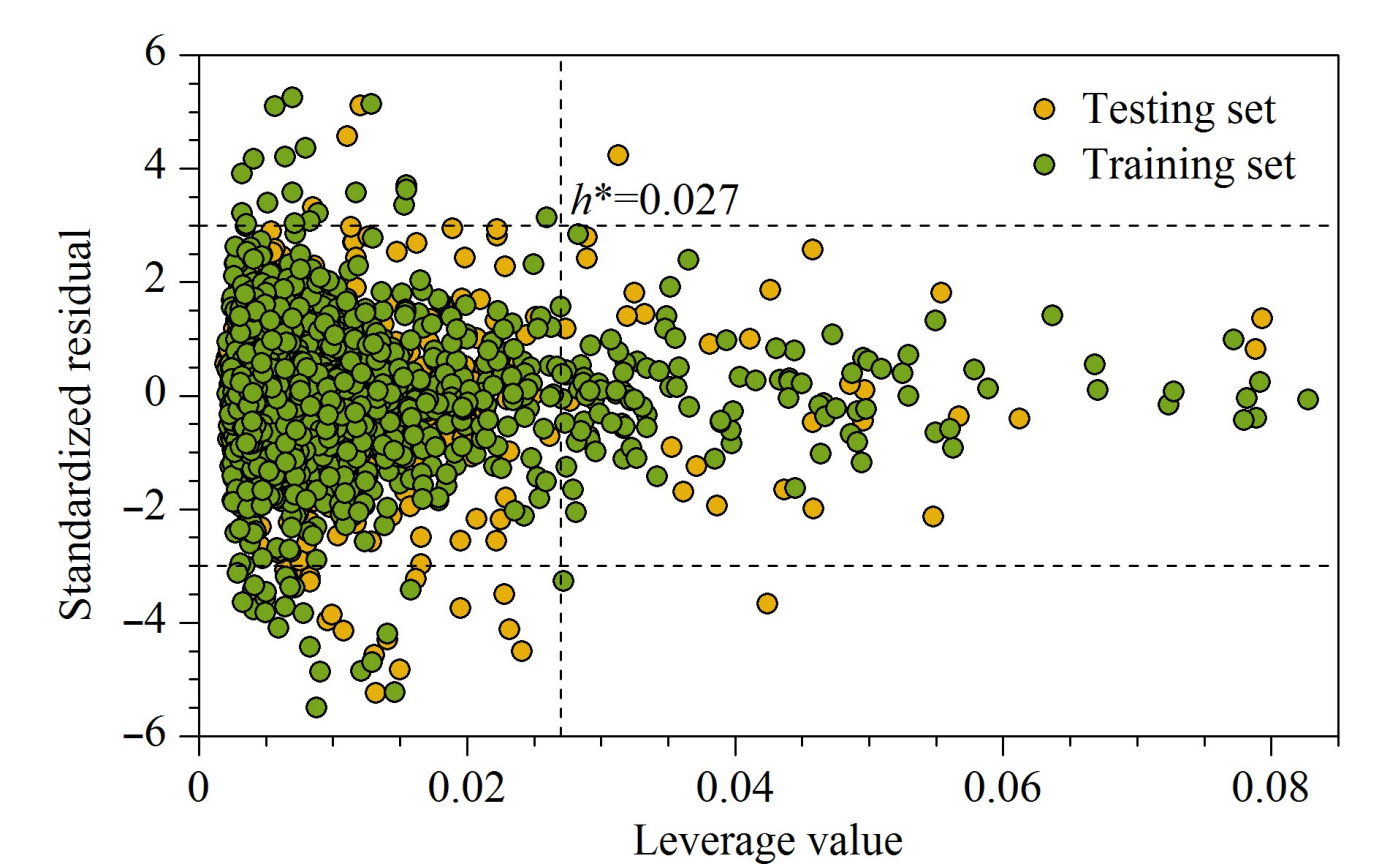
投票回归模型的Williams图

### 2.5 模型比较

本研究数据集包含2499种化合物在8种类型色谱柱上的RI数据,模型输入为化合物的分子结构特征及固定相极性的独热编码,输出为化合物在该固定相上的RI预测值。表4列出了本研究与之前研究的模型预测性能对比。

**表4:** 集成学习模型和以前模型的比较

Order	Method	Stationary phases	Number	Training				Testing			Ref.
				*R*^2^	RMSE	MAE		*R*^2^	RMSE	MAE	
1	VR	five polar stationary phases	4183	0.99	101.85	23.60		0.97	107.44	75.28	this study
2	GNN	SSNP	29518	-	-	11.80		0.99	-	30.92	[11]
		SNP	14033	-	-	23.33		0.99	-	42.41	
		SP	7052	-	-	45.46		0.95	-	84.34	
3	GNN	non polarity	94183	-	20.69	-		-	57.90	-	[12]
4	PLR	non polarity	90	-	-	-		0.99	17.40	-	[10]
5	-	strong polarity	1179	0.83	170.90	124.20		0.90	132.90	102.70	[8]

MAE: mean absolute error; GNN: graph neural networks; PLR: partial least squares regression; SSNP: semi-standard nonpolar; SNP: standard nonpolar; SP: standard polar.

本研究所建立的模型适用于化合物在5种不同极性固定相上的RI预测,扩展了预测范围,有效解决了单一极性固定相预测模型因极性变化导致RI预测准确性不足的问题,极大地提升了模型在气相色谱靶标和非靶标筛查工作中的适用性和灵活性,并将环境污染物纳入数据集中,为环境分析监测提供了有力支持,最后进行应用域表征,提供模型使用的重要参考依据。

然而,未来该模型的潜力还有待进一步挖掘。受限于相关研究的数据集未公开,本研究建立模型可使用数据量较为有限。同时可以考虑使用图神经网络或其他机器学习方法,以更充分地挖掘和利用化合物间复杂的相互作用和结构信息,提高模型预测精度。

## 3 结论

本研究计算得到化合物1维和2维分子结构特征,根据McReynolds常数将固定相进一步划分为5类极性特征,耦合分子结构特征和固定相分类特征,应用9种个体机器学习算法和集成学习算法,建立了2499种化合物在5种极性固定相上的RI预测模型。该模型能适应多种极性固定相RI的预测,对于提高气相色谱靶标和非靶标分析的效率具有重要意义和价值。未来可以继续扩充数据集,引入深度学习技术,以进一步挖掘化合物结构信息,提升模型的泛化能力和预测精度。
